# OP47 New Health Technology Assessment Framework For Digital Health Technologies

**DOI:** 10.1017/S0266462324001090

**Published:** 2025-01-07

**Authors:** Joan Segur-Ferrer, Carolina Moltó-Puigmartí, Roland Pastells Peiró, Rosa Maria Vivanco-Hidalgo

## Abstract

**Introduction:**

Experts emphasize the necessity of reconsidering the approaches used in the health technology assessment (HTA) of digital health technologies (DHTs) to adequately address their particular characteristics. In this context, the Agency for Health Quality and Assessment of Catalonia was commissioned to develop a specific framework for the assessment of DHTs in collaboration with the agencies that constitute the Spanish Network of Agencies for Assessing National Health System Technologies and Performance (RedETS).

**Methods:**

To accomplish the assignment, a combination of methodologies was used. To establish the domains and dimensions, an international survey, a scoping review of the literature, and a thematic analysis were executed. To determine the evidence standards that DHTs must achieve according to the risk classification derived from their use, the updated version of the evidence standards framework (ESF) from the National Institute for Health and Care Excellence (NICE) was adapted to the Spanish context. National experts in the fields of HTA and DHTs collaborated on different tasks to develop the framework.

**Results:**

The framework encompasses three distinct sections. The first section provides an introduction, detailing a DHT classification system that is based on the system proposed by NICE in their ESF. Following this, Section A elaborates on a comprehensive set of 13 domains, 41 dimensions, and nine sub-dimensions, offering a detailed description of each. Section B consists of the adaptation of the 21 evidence standards from the NICE ESF to the Spanish context. Of note, a clear link was established between the classification system, the domains, dimensions, sub-dimensions, and the evidence standards.

**Conclusions:**

The resulting framework was published in November 2023. Since its publication, it has become the reference framework for the HTA of DHTs within the Spanish National Health System, primarily by the HTA agencies that constitute RedETS. The document will be periodically reviewed and, if necessary, adapted to align with emerging technologies and changes in legislation.

